# 3D-printed copper-containing tailored titanium alloys with corrosion resistance, biocompatibility, and anti-inflammatory properties for enhanced guided bone regeneration

**DOI:** 10.3389/fbioe.2025.1647678

**Published:** 2025-08-21

**Authors:** Lan Luo, Quan Zhong, Zi-Qin Chen, Xiao-Hong Wu, Shu-Man Li, Zhen-Zhu Xue, Yan-Jin Lu, Kai Luo, Wei Zhao

**Affiliations:** ^1^ Institute of Stomatology and Laboratory of Oral Tissue Engineering, School and Hospital of Stomatology, Fujian Medical University, Fuzhou, China; ^2^ Fujian Key Laboratory of Oral Diseases and Fujian Provincial Engineering Research Center of Oral Biomaterial and Stomatological Key laboratory of Fujian College and University, School and Hospital of Stomatology, Fujian Medical University, Fuzhou, China; ^3^ Department of Stomatology, Fujian Provincial Geriatric Hospital, Fuzhou, China; ^4^ Key Laboratory of Opto-Electronic Science and Technology for Medicine of Ministry of Education, College of Photonic and Electronic Engineering, Fujian Normal University, Fuzhou, China

**Keywords:** Ti-Cu alloy, corrosion resistance, biocompatibility, anti-inflammatory, osteogenesis

## Abstract

**Introduction:**

Guided bone regeneration (GBR) serves as a critical technique in dental implantology, relying heavily on barrier membranes for successful alveolar bone augmentation. Titanium mesh, widely utilized in GBR procedures, faces a high exposure rate that leads to infections and compromised clinical outcomes. While 3D-printed personalized meshes have reduced exposure rates, infection risks persist, necessitating the development of bioactive solutions.

**Methods:**

In this study, selective laser melting (SLM) was employed to fabricate copper-bearing titanium meshes using Ti-*x*Cu powders (x=0, 4, 6, 8 wt%). This investigation systematically evaluated the effects of copper content on corrosion resistance, biocompatibility, osteogenic potential, and anti-inflammatory properties of the Ti-*x*Cu alloys.

**Results:**

Microstructural analysis revealed that increasing copper content enhanced Ti_2_Cu precipitation within the α-Ti matrix. While increased copper content did not compromise corrosion resistance, it resulted in higher copper ion release concentrations. Antibacterial assays demonstrated that alloys with copper content exceeding 4 wt% exhibited >90% bacterial reduction against S. aureus and *E. coli*. *In vitro* studies showed that Ti-6Cu optimally promoted osteoblast proliferation and upregulated osteogenic genes (*Alp, Col-1*). Furthermore, Ti-6Cu upregulated anti-inflammatory factors (*Il-10, Arg-1*) while downregulating inflammatory factors (*Tnf-α, Il-6*).

**Conclusion:**

The study established SLM-treated antibacterial Ti-6Cu alloy exhibited favorable biological activity, demonstrating promising potential for application in regeneration scaffolds.

## 1 Introduction

Implant dentistry is the preferred treatment for tooth loss. In cases of severe bone atrophy, bone reconstruction is essential for implant stability and soft tissue restoration (Li et al., 2021). Currently, the main clinical strategy to increase alveolar bone volume is guided bone regeneration technology (GBR), owing to its ease of clinical application and reliability (Tolstunov et al., 2019). The barrier membrane technique serves as a critical technical approach in GBR procedures, where membrane performance directly determines the clinical outcome. Titanium mesh has been extensively utilized in alveolar bone reconstruction due to its excellent mechanical strength, corrosion resistance, and biocompatibility. Titanium alloys have become the main materials in the dental and orthopedic fields (Marin and Lanzutti, 2023; Xie et al., 2020). However, exposure of the titanium mesh remains a prevalent postoperative complication, with an overall exposure rate of 44% (95% CI: 0.30 ∼ 0.58) (Zhou et al., 2022). Possible factors contributing to the exposure include wound soft tissue rupture, insufficient thickness of residual bone walls, infection, and sharp mechanical trauma.

The advent of the three-dimensional (3D) printing technology has revolutionized clinical practice, particularly through advanced manufacturing techniques like selective laser melting (SLM) (Chen et al., 2021; Di Spirito et al., 2024). Driven by digital innovation, SLM enables the fabrication of patient-specific medical devices that anatomically conform to individual tissue defects with increased production efficiency and cost-effectiveness (Scribante et al., 2023; Cui et al., 2022). In contrast to traditional titanium meshes, 3D-printed personalized meshes exhibit an exposure rate of 31%, significantly lower than the 51% rate observed for traditional meshes (Zhou et al., 2022). This finding highlights that patient-specific 3D-printed titanium meshes not only minimize the risk of exposure but also maintain effective bone regeneration and the minimally invasive aspect of surgical interventions.

Despite these improvements, mesh exposure persists as a critical clinical challenge affecting approximately one-third of cases. Pathogen colonization on exposed surfaces frequently precipitates localized infections, which in turn can impair the efficacy of regenerative therapies. Currently, the cornerstone therapeutic strategy for clinical infections in titanium mesh GBR remains surgical debridement combined with mesh replacement and adjuvant antibiotic therapy (Arciola et al., 2018). Growing antibiotic resistance and increasing clinical needs for bioactive bone regeneration have accelerated the development of antimicrobial-functionalized implants (Jacobs et al., 2020). CpTi and α+β Ti alloys (e.g., Ti-6Al-4V) are common orthopedic implant materials. However, their high elastic modulus compared to natural bone can cause stress shielding, hindering osseointegration. Ti-6Al-4V may also release cytotoxic Al/V ions (Calazans Neto et al., 2024). β-type Ti alloys show promise as a viable solution (Liu et al., 2023). In antibacterial β-type Ti alloys, copper (Cu) occupies a distinctive position compared to other antimicrobial elements such as silver (Ag) and zinc (Zn) (Yi et al., 2021). Researchers have crafted medical alloys with comprehensive and long-lasting self-antibacterial capabilities, with copper-enriched medical metals emerging as especially noteworthy (Zhang et al., 2022). Copper is a critical trace element for humans, ranking immediately after iron and zinc, and plays a crucial role in physiological processes such as cell metabolism and catalytic activities (Lu et al., 2024). The average adult body contains roughly 150 mg of Cu, with approximately 60% located in muscles and bones, 10% in the bloodstream, and the remainder in enzymes (Hoppe et al., 2011). Notably, Cu ions are known for their potent antibacterial effects, with historical applications in wound and dermatological infections (Yang et al., 2024).

Building on this, scientists have alloyed Cu into titanium alloys to benefit from the gradual release of ions for antibacterial and biological purposes, resulting in the development of Ti-Cu (Zhe et al., 2021; Liu et al., 2022), Ti6Al4V-Cu (Zhang et al., 2024a), Ti-Zr-Cu (Alkindi et al., 2023), etc. Studies suggest that Ti-Cu alloys possess not only potent antibacterial effects and significant osteogenic potential but also exhibit enhanced resistance to wear and corrosion (Arroussi et al., 2023; Liang et al., 2025). These advantageous attributes position Ti-Cu alloys as promising candidates for next-generation biofunctional titanium meshes in biomedical applications. However, systematic investigations comparing corrosion resistance, antibacterial efficacy, inflammatory response, and osteogenic potential in Ti-*x*Cu alloys fabricated by SLM with varying Cu mass fractions have not been sufficiently conducted, particularly regarding their microstructure-property relationships under additive manufacturing conditions.

**FIGURE 1:**
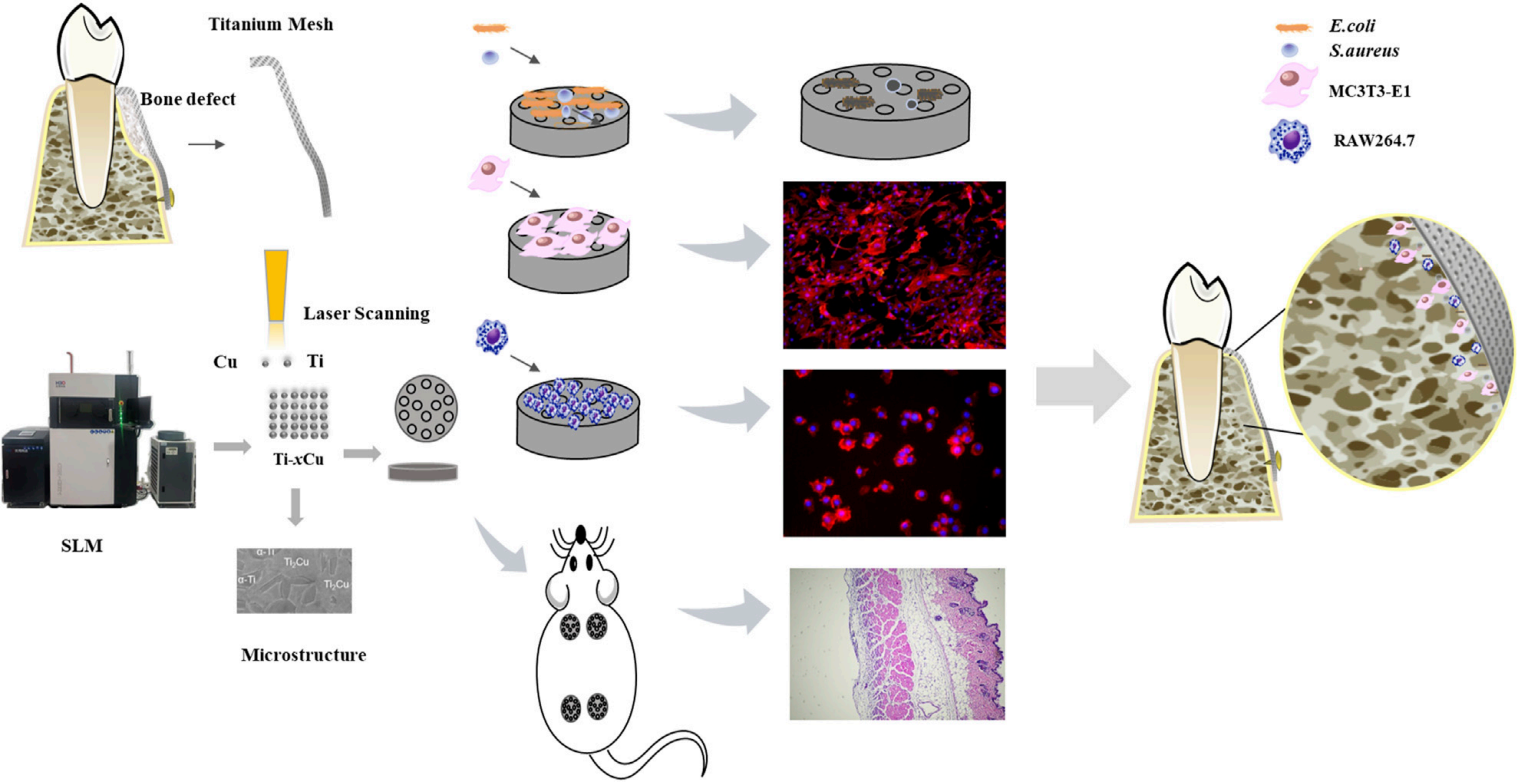
Schematic diagram of the biological properties of Ti-*x*Cu alloys.

In this study, SLM is employed to fabricate Cu-bearing titanium mesh via Ti-*x*Cu (*x* = 0, 4 wt%, 6 wt%, 8 wt%) powders. The study investigates the effect of Cu content on the corrosion resistance, biocompatibility, osteogenic potential, and anti-inflammatory nature of the Ti-*x*Cu alloys shown in Figure 1. The objective is to lay the groundwork for the clinical application of personalized 3D-printed titanium meshes with antibacterial activity.

## 2 Experimental details

### 2.1 Material fabrication

**FIGURE 2:**
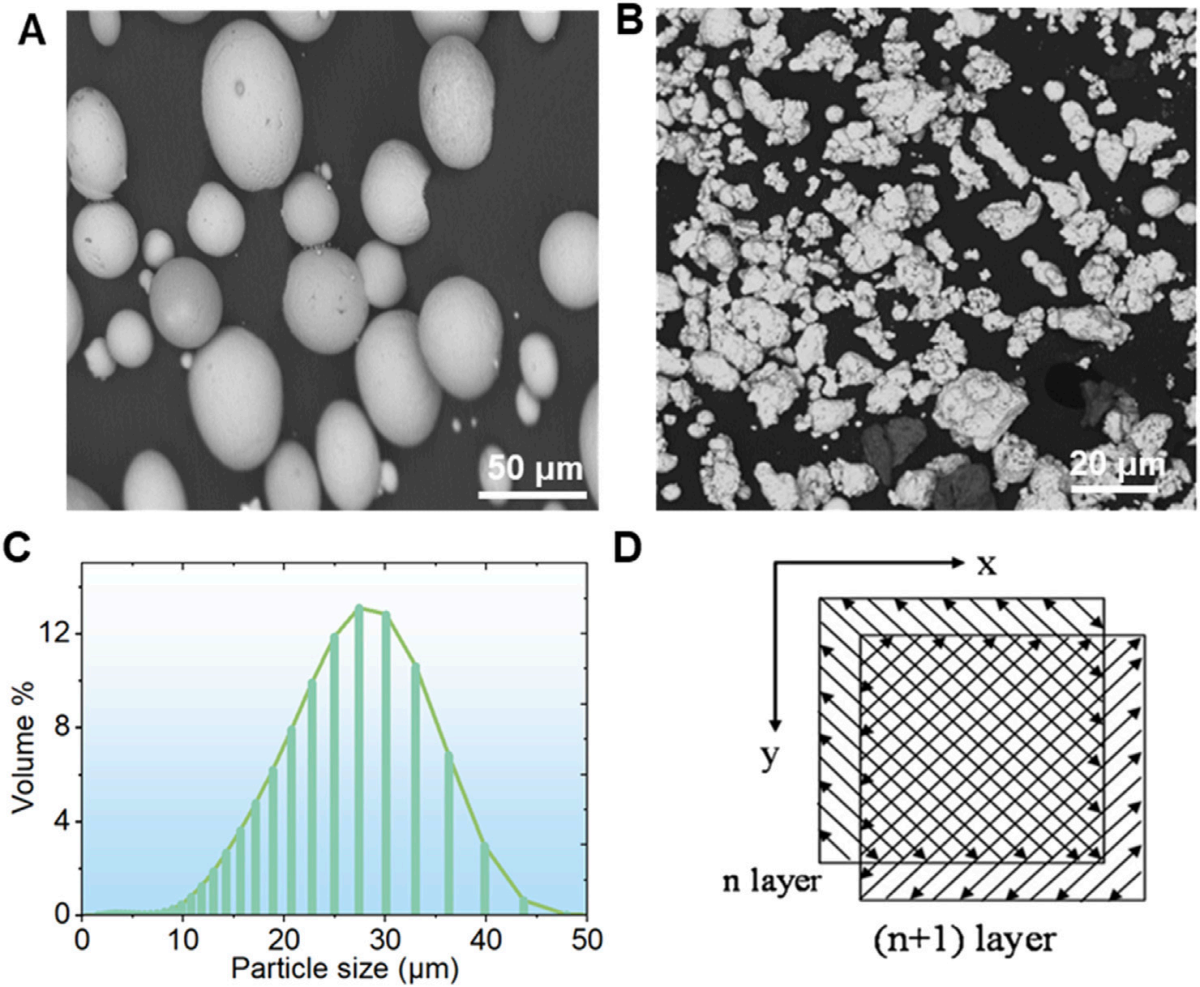
The morphology of the **(A)** Ti and **(B)** Cu powders; **(C)** the particle size distribution of Ti powders and laser scanning pattern; **(D)** the laser scanning strategy for SLM.

Ti-*x*Cu (where *x* equals 0, 4 wt%, 6 wt%, or 8 wt%) bulk alloys were fabricated by mixing pure copper powders with contents at 0 wt%, 4 wt%, 6 wt%, or 8 wt% into commercial-grade pure titanium powder (99.99%). A planetary ball mill was employed to mix the powders at a rotational speed of 400 rpm to ensure thorough and uniform mixing of the powders. The shapes of the Ti and Cu powders are depicted in Figures 2A,B. The titanium powder predominantly exhibits a spherical form in Figure 2A, whereas the copper powder shows an irregular shape in Figure 2B. The pure titanium powder has a size of 15–45 µm in Figure 2C, while the pure copper powder has a particle size of less than 10 µm. Following a 1-h thorough mixing of the titanium and copper powders, Ti-*x*Cu alloys with a relative density exceeding 99.5% were manufactured using the selective laser melting (SLM) technique. The resulting alloys were designated as Ti, Ti-4Cu, Ti-6Cu, and Ti-8Cu. SLM parameters with a scanning speed of 600 mm/s, 85 W of laser power, and 110 μm of space width were used. During the SLM process, a scanning pattern characterized by a zigzag trajectory with cross-hatching was employed. The orientation of the scanning strategy was consistently rotated by 90° when transitioning from one layer to the subsequent layer in Figure 2D.

### 2.2 Microstructural analysis

Post-metallographic observations, samples were subjected to analysis with a scanning electron microscope (SEM, SU8010, Japan) coupled with an energy-dispersive spectrometer (EDS) to analyze their microstructure and elemental distribution across different phases. The alloy’s phase composition was identified via a D/MAX-2500PC X-ray diffractometer, equipped with a Cu-target Kα radiation source. The scan was conducted at a rate of 5° per minute over an angular range of 10°–80°. The acquired X-ray diffraction (XRD) data were subsequently processed using Jade 5.0 software to generate the corresponding XRD patterns.

### 2.3 Electrochemical test

The corrosion resistance of Ti-*x*Cu was assessed using a conventional three-electrode workstation (Gamry REFERENCE 600+, USA). A saturated calomel electrode (SCE) functioned as the reference electrode (RE), a platinum electrode acted as the counter electrode (CE), and the sample, which had a polished surface area of 1 cm^2^ post-encapsulation, served as the working electrode (WE). Electrochemical measurements were conducted in 0.9% NaCl at 37°C ± 1°C. After 1 h stabilization, the electrochemical impedance spectroscopy (EIS) was performed (1–2×10^5^ Hz), followed by potentiodynamic polarization (−0.5–1.5 V, 0.5 mV/s), with triplicate measurements for each sample.

### 2.4 Immersion test

In line with ISO 10271:2001, cylindrical samples measuring Φ10 mm × 10 mm (with a surface area exceeding 10 cm^2^) were placed in 0.9 wt% NaCl solution at an elution ratio of 1 cm^2^/mL and incubated at 37°C for 7 days, with triplicate samples for each group. Then, the immersed solution was analyzed for elemental concentration using an inductively coupled plasma atomic emission spectrometer (ICP-AES, Ultima 2).

### 2.5 Culture of MC3T3-E1 and RAW cells

The RAW264.7 murine macrophages and MC3T3-E1 murine osteoblasts (Cell Bank of Chinese Academy of Sciences) were cultured in DMEM and α-MEM (supplemented with 10% FBS, HyClone, USA), respectively, at 37°C/5% CO_2_. Cells were passaged every 2–3 days. For experiments, macrophages (1 × 10^5^ cells/well) and osteoblasts (2 × 10^4^ cells/well) were seeded on Ti-*x*Cu alloys in 24-well plates.

### 2.6 Cellular proliferation

Ti*-x*Cu alloys (n = 6) were placed into 24-well culture plates. Cells were then seeded onto the surfaces of these materials to evaluate their proliferative capacity at multiple time intervals using the CCK-8 assay. The CCK-8 reagent (Dojindo, Japan) was administered to each well following the protocol outlined in the CCK-8 kit manual. Following incubation of 1 day, 3 days, 5 days, and 7 days at 37°C, the optical density (OD) at a wavelength of 450 nm for each well was determined utilizing a spectrophotometric microplate reader using an iMark microplate reader (iMark, Bio-Rad Laboratories, USA).

### 2.7 Live/dead cell staining assay

After culturing the MC3T3-E1 cell line on the surfaces of various material groups, the samples were carefully transferred to a new 24-well plate using sterile forceps and washed three times with PBS. A staining solution containing calcein-AM and propidium iodide (PI) (Beyotime, China) was then added, followed by incubation at 37°C for 15 min. Subsequently, the samples were rinsed three times with PBS and imaged under a fluorescence microscope. Red fluorescence indicated dead cells, whereas green fluorescence represented live cells. Cell viability was calculated as the percentage of live cells relative to the total cell count.

### 2.8 Immunofluorescence staining

To evaluate cell adhesion and spreading across various samples, rhodamine phalloidin and DAPI staining were employed to delineate the cytoskeleton and nuclei of the cells, respectively. Ti-*x*Cu alloys were placed in 24-well plates, and cells were seeded onto these surfaces. The samples were fixed with 4% paraformaldehyde for 20 min and then incubated in the dark at 37°C for 30 min. Subsequently, the samples were subjected to DAPI staining for 5 min followed by three PBS washes. The cytoskeleton and nuclei of MC3T3-E1 cells were examined using a fluorescence microscope (Olympus, Japan), with images captured for blue emissions (under 400–440 nm excitation) and red emissions (under 510–550 nm excitation).

### 2.9 Alkaline phosphatase staining

According to international standard ISO 10993-12 (Zhang et al., 2016), the immersed samples were stored in air at 37°C and 5% CO_2_ with a specific surface area-to-volume ratio of 1.25 cm^2^/mL for 72 h to prepare the extracts. MC3T3-E1 cells were inoculated in 24-well plates.

### 2.10 Real-time PCR

RAW264.7 macrophages (1 × 10^5^ cells/well, n = 4) were cultured on Ti-*x*Cu for 6 h/72h, MC3T3-E1 osteoblasts (n = 4) were cultured on Ti-*x*Cu substrates in osteogenic induction medium for 7 days. Total RNA was extracted and reverse-transcribed using a PrimeScript RT kit (Takara, Japan). Quantitative PCR was performed on a LightCycler480 system (Roche) with SYBR Green mix, using NCBI-derived primers (Table 1; synthesized by Shanghai Shenggong Biotech). Gene expression was normalized to GAPDH via the 2^−ΔΔCT^ method.

**TABLE 1:** The primer sequence.

Gene	Forward primer sequence (5′–3′)	Reverse primer sequence (5′–3′)
*Gapdh*	TGG​AAA​GCT​GTG​GCG​TGA​TG	TAC​TTG​GCA​GGT​TTC​TCC​AGG
*Alp*	TGA​GCG​ACA​CGG​ACA​AGA​AG	CTG​GTA​GTT​GTT​GTG​AGC​GTA​ATC
*Col-1-*	AGC​ACG​TCT​GGT​TTG​GAG​AG	GAC​ATT​AGG​CGC​AGG​AAG​GT
*Runx2*	AAG​GCA​CAG​ACA​GAA​GCT​TGA	AGG​ACT​TGG​TGC​AGA​GTT​CAG
*Opn*	AGC​AAG​AAA​CTC​TTC​CAA​GCA​A	GTG​AGA​TTC​GTC​AGA​TTC​ATC​CG
*Tnf-α*	GCC​GAT​GGG​TTG​TAC​CTT​GT	TCT​TGA​CGG​CAG​AGA​GGA​GG
*Il-1β*	GAA​ATG​CCA​CCT​TTT​GAC​AGT​G	TGG​ATG​CTC​TCA​TCA​GGA​CAG
*Arg-1*	CAG​CAG​AGG​AGG​TGA​AGA​GTA	TAG​TCA​GTC​CCT​GGC​TTA​TGG
*Il-6*	CTG​CAA​GAG​ACT​TCC​ATC​CAG	AGT​GGT​ATA​GAC​AGG​TCT​GTT​GG
*Il-10*	CTT​ACT​GAC​TGG​CAT​GAG​GAT​CA	GCA​GCT​CTA​GGA​GCA​TGT​GG

### 2.11 Antibacterial tests

*Escherichia coli* (*E. coli*) and *Staphylococcus aureus* (*S. aureus*) suspensions adjusted to a McFarland standard of 0.25 were co-cultured with Ti-*x*Cu alloys, respectively. After 24-h incubation, 100-μL aliquots were taken from each culture and evenly spread onto nutrient agar plates. These plates were then incubated at 37°C in a humidity-controlled chamber with a humidity of 90% for 24 h. Once the incubation was complete, the number of bacterial colonies on each plate was counted. The antibacterial efficacy was evaluated using the following formula:

$$\text{Antibacterial rate}=\left(1-\frac{\text{Experimental}\;\text{group}\;\text{colony}\;\text{count}\;}{\text{Control}\;\text{group}\;\text{colony}\;\text{count}}\right)\times 100\%$$

### 2.12 *In vivo* study

Twelve clean-grade, 6-week-old male ICR mice weighing 20–30 g were used in the study. The mice were obtained from Shanghai Slack Laboratory Animal Co., Ltd. (Production License No. SCXK 2012-0002). This animal experiment was approved by the Experimental Animal Ethics Committee of Fujian Medical University (Approval No. IIACUC FJMU 2023-0184). Following a 7-day acclimation period with *ad libitum* access to food and water, mice underwent air pouch establishment. Dorsal fur was shaved, and 4 mL sterile air was injected subcutaneously on days 0 and 4 to induce pouch formation. Successful pouch development was confirmed on day 5 by the absence of inflammatory signs. On day 7, animals were anesthetized (1% sodium pentobarbital, 1 mL/100 g body weight), and implants were aseptically inserted into the pouches with subsequent wound closure. After 7 days post-implantation, major organs were harvested, fixed in 4% paraformaldehyde, and embedded in paraffin. Tissue sections (10 μm) were prepared for hematoxylin and eosin (H&E) staining and histological examination.

### 2.13 SPSS statistics

All displayed data were represented as mean ± standard deviation (SD). Statistically significant differences were analyzed by one-way analysis of variance (ANOVA) and Dunnett’s t-tests. *P* < 0.05 was considered significant. *P < 0.05, **P < 0.01, ***P < 0.001.

## 3 Results

### 3.1 Microstructure

**FIGURE 3:**
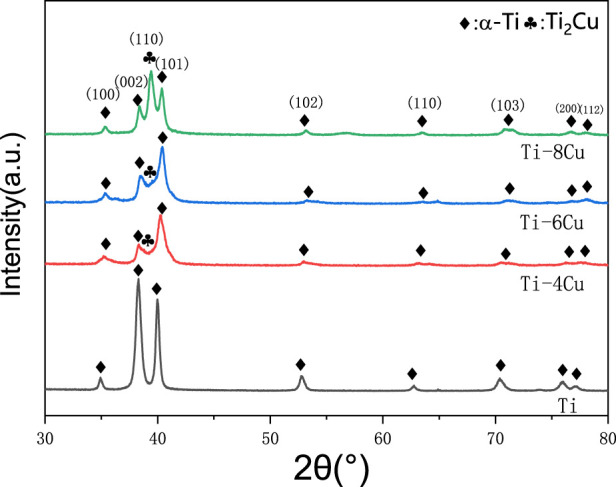
XRD pattern for Ti-*x*Cu alloys.

As depicted in Figure 3, the XRD patterns for the Ti-*x*Cu (*x* = 0, 4 wt%, 6 wt%, 8 wt%) alloys exhibit a high similarity among the four groups. Predominantly, the pure Ti- and Cu-bearing Ti alloys consist of the α phase. In Ti-*x*Cu alloys, the intensity of the diffraction peak for the Ti_2_Cu phase increases with the addition of copper. When the copper content reaches 8 wt%, the diffraction peak for the Ti_2_Cu phase becomes the strongest. Additionally, XRD patterns demonstrate that the diffraction peak around 40° shifts to higher angles as the copper content increases. This is because with the increase of copper content, the diffraction peak near 40° in the XRD pattern (Figure 3) shifts to the right. The observed phenomenon primarily results from lattice distortion induced by copper incorporation into the titanium matrix. Given copper’s smaller atomic radius compared to titanium, its dissolution in the α-Ti lattice causes lattice contraction, consequently reducing interplanar spacing (d-spacing). According to Bragg’s law (nλ = 2*d* sinθ), a reduced d-value increases the diffraction angle (θ), shifting the peak toward higher angles. At higher Cu content (≥4 wt%), Ti_2_Cu precipitation further distorts the lattice. The tetragonal Ti_2_Cu structure, differing from hexagonal α-Ti, additionally influences peak positions.

**FIGURE 4:**
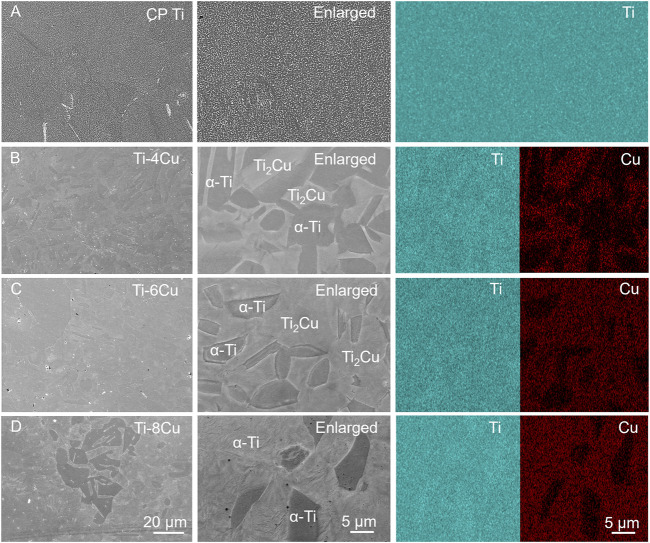
The SEM images along with their respective EDS spectra for the microstructures of **(A)** Ti and **(B)** Ti-4Cu; **(C)** Ti-6Cu; **(D)** Ti-8Cu alloys.

Figure 4 displays the SEM images along with their respective EDS spectra for the microstructures of Ti and the Ti-4Cu, Ti-6Cu, and Ti-8Cu alloys. In Figure 4A, the Ti microstructure is characterized by small α-phase grains that are consistently dispersed within the matrix. Figures 4B–D show that after alloying the Cu into Ti, the resulting microstructure of the alloy is dominated by lamellar Ti_2_Cu (marked as bright regions in the enlarged image) and an α-Ti matrix (marked as dark regions in the enlarged image). A significant observation is the progressive enlargement of the Ti_2_Cu area and the concurrent increase in grain size with increased Cu content in the matrix. Notably, at 8 wt%Cu content, the amount of Ti_2_Cu surpasses that of the matrix. In this study, the laser power of 85 W provides sufficient energy density for complete powder melting without excessive penetration, ensuring molten pool stability. These parameters maintain a cooling rate (approximately 10^5^–10^6^ K/s), which suppresses macroscopic segregation of Cu elements and promotes the uniform distribution of nanoscale Ti2Cu precipitates. However, at 8 wt% Cu, the molten pool stability decreases owing to Cu’s high latent heat and altered surface tension, resulting in heterogeneous solidification and a broader Ti_2_Cu size distribution. This phenomenon is consistent with the solute enrichment mechanism revealed in the study by Zhang et al. (2019). The study shows that high Cu content intensifies constitutional supercooling at the solidification front, thereby affecting the nucleation and growth of precipitates.

### 3.2 Electrochemical test

**FIGURE 5:**
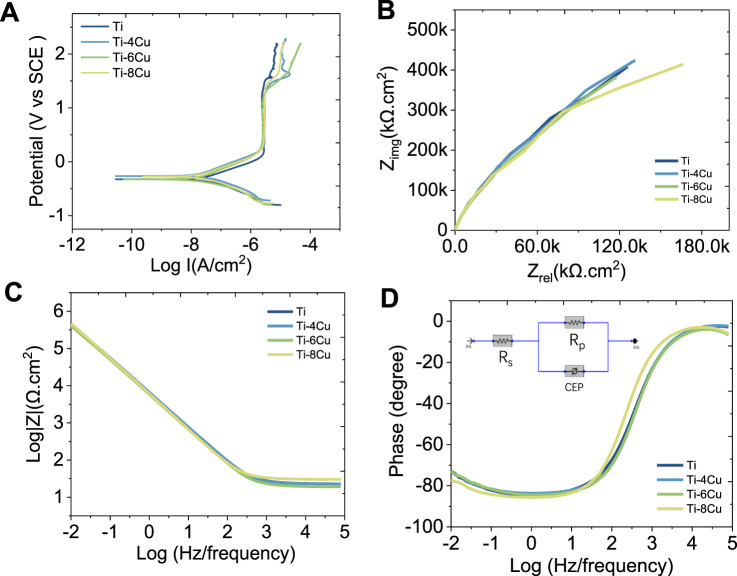
**(A)** Potential dynamic polarization curves of Ti-*x*Cu alloys; **(B)** Nyquist plots; **(C)** Bode impedance plots; **(D)** Bode phase plots of Ti-*x*Cu alloys recorded in 0.9 wt% NaCl solution and corresponding equivalent circuit inserted in Figure 5D.

Figure 5A depicts the potentiodynamic polarization curves of Ti-*x*Cu alloys in a 0.9 wt% NaCl solution at 37°C, respectively. The corresponding corrosion parameters are listed in Table 2. The curves for the four groups are notably consistent, each showing a sequence of activation and passivity, indicative of a similar electrochemical corrosion behavior. The transition from the cathodic to anodic potential signifies the alloy’s corrosion potential (E_corr_), after which the curve enters the passive phase, in which the current density increases linearly with the increase in corrosion potential. Alloying Cu into the pure Ti leads to a general upward shift in the curves compared to pure Ti, which corresponds to a decrease in the I_corr_. The I_corr_ values for Ti and the Ti-4Cu, Ti-6Cu, and Ti-8Cu alloys are 3.76 × 10^−8^, 1.93 × 10^−8^, 1.37 × 10^−8^, and 2.84 × 10^−8^ A cm^2^, respectively. A decreasing I_corr_ is found with higher Cu content. As the Cu reaches 8 wt%, the I_corr_ increases to 2.84 × 10^−8^ A cm^2^. Despite this increase, the Ti-6Cu alloy still exhibits a lower corrosion current than the Ti alloy.

**TABLE 2:** Electrochemical data from potential dynamic polarization tests.

Samples	I_corr_/A.cm^2^ × 10^-8^	E_corr_/mV
Ti	3.76 ± 0.11	−325 ± 1.95
Ti-4Cu	1.93 ± 0.28	−265 ± 2.88
Ti-6Cu	1.37 ± 0.14	−327 ± 2.76
Ti-8Cu	2.84 ± 0.26	−275 ± 2.0

Figure 5B presents the Nyquist diagrams of Ti and the Ti-4Cu, Ti-6Cu, and Ti-8Cu alloys. All curves exhibit a semicircular shape, suggesting single-layer film corrosion. The capacitive arc reflects charge transfer resistance, which is a measure of the electrode reaction’s velocity and a determinant of the passive film’s corrosion resistance. Comparing the radii of the capacitive arcs in the Nyquist diagrams reveals the stability and corrosion resistance of the Ti-*x*Cu alloys. That is, a larger arc radius corresponds to higher impedance and stronger corrosion resistance. As observed in Figure 5B, the capacitive arc radius does not change significantly with increasing Cu content, suggesting that the addition of Cu does not adversely affect the stability of the passive film. Figures 5C,D present the Bode diagrams for the Ti-*x*Cu alloys. In the high-frequency range (1–100 kHz), the phase angle of all alloys decreases as frequency increases, while the impedance value |Z| remains largely unchanged, indicating that impedance at high frequencies is linked to the solution resistance. In the low-frequency range (<100 Hz), as frequency decreases, the phase angle of all alloys approaches 90°. A phase angle near 90° typically signifies a stable, dense passive film. At 0.01 Hz, Ti-*x*Cu alloys exhibit phase angles similar to pure Ti, suggesting Cu addition minimally affects passive film durability or corrosion resistance. Additionally, the Bode plot’s slope of ∼ −1 (0.1–1,000 Hz) indicates capacitive behavior consistent with a single-layer passive film, further confirming the formation of a dense protective layer.

The Bode and Nyquist diagrams indicate that a single-layer film forms on the surface. An equivalent electrical circuit (EEC) featuring a single constant-phase element (CPE) is used to model this corrosion, as inserted in Figure 5D. The CPE, denoted by Q, describes the deviation of the double-layer parameters at the electrode–solution interface from capacitance C and is independent of frequency. It is related to the surface roughness, composition, porous electrodes, adsorption reactions, and other material conditions. The admittance and impedance of the CPE are given by

$$Z_{\text{CPE}}=\frac{1}{Y_{0}}{\left(j\omega \right)}^{-n}$$

where Y_0_ is the CPE’s magnitude, j is the imaginary unit, and n is an exponent less than 1 for the CPE. A decreasing n value closer to 1 indicates fewer pores in the passive layer, signifying a denser layer. In the equivalent circuit, R_s_ and R_p_ represent the resistance of the electrolyte and the passive layer, respectively, while Q signifies the capacitance of the passive film. Table 3 lists the electrochemical parameters of the Ti-*x*Cu alloys derived from the equivalent circuit model. The polarization resistance R, of Ti is 1.94 × 10^6^ Ω cm which increases with Cu content to 2.34 x 10^6^ Ω cm for Ti-4 Cu. A higher polarization resistance indicates better corrosion resistance. However, R_p_ slightly decreases when the copper content reaches 6 wt% and 8 wt%. Additionally, the n values for Ti-*x*Cu are very close, suggesting a dense passive layer and good corrosion resistance for Cu-bearing Ti alloys.

**TABLE 3:** Equivalent circuit parameters calculated by fitting EIS data.

Sample	*R*_s_ (Ω·cm^2^)	*R*_p_ (10^6^ Ω·cm^2^)	*Q*_p_±0.78 (10^−6^ Ω^-1^·s^-n^·cm^-2^)	*n*_p_
Ti	22.11 ± 1.32	1.94 ± 0.28	21.12 ± 1.48	0.92 ± 0.01
Ti-4Cu	23.59 ± 2.40	2.34 ± 0.32	28.39 ± 2.54	0.93 ± 0.02
Ti-6Cu	20.93 ± 1.73	1.88 ± 0.20	31.34 ± 2.13	0.93 ± 0.01
Ti-8Cu	24.83 ± 2.64	1.69 ± 0.27	27.42 ± 1.56	0.92 ± 0.03

### 3.3 Immersion test and antibacterial test

**FIGURE 6:**
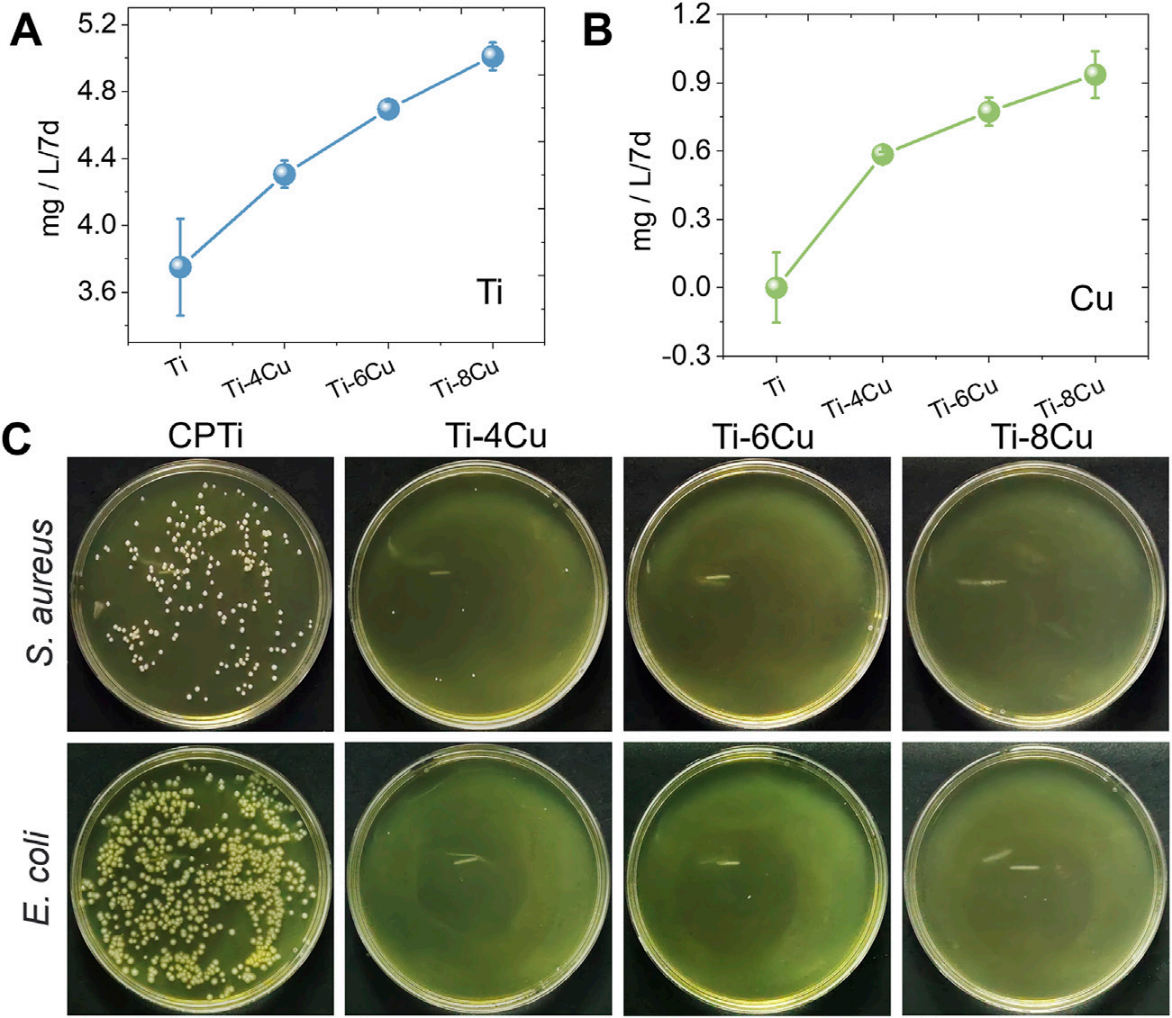
The release pattern of **(A)** Ti and **(B)** Cu ions from Ti-*x*Cu alloys, and **(C)** the antibacterial activity against Staphylococcus aureus and E. coli after being cultured with alloys for 24 h.

Figures 6A,B illustrate the release pattern of Ti and Cu ions in a 0.9 wt% NaCl solution, respectively. The profiles clearly show that Ti and Cu ions increase with higher concentrations of Cu content. To assess the antibacterial performance of Ti-*x*Cu alloys, these samples were cultured with *E. coli* and *S. aureus* for 24 h, respectively, as shown in Figure 6C. The data from Figure C demonstrate that pure Ti has a high density of bacterial colonies, while Ti-4Cu, Ti-6Cu, and Ti-8Cu show a marked decrease in colony count. This suggests that Ti-4Cu, Ti-6Cu, and Ti-8Cu possess superior antibacterial properties. Meanwhile, the antibacterial rates for these alloys are above 99%.

### 3.4 *In vitro* biocompatibility evaluation of Ti-*x*Cu alloys

**FIGURE 7:**
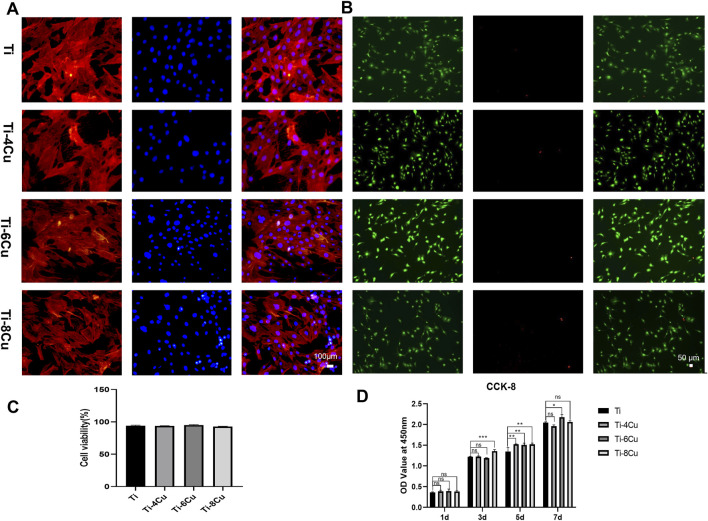
**(A)** The adhesion morphology of MC3T3 on Ti-*x*Cu alloys after 3 days of co-culturing; **(B)** Live/dead cell staining; **(C)** cell viability analysis; **(D)** the cell viability across various groups for 1 day, 3 days, 5 days, and 7 days.

Figure 7A shows MC3T3 cell adhesion on Ti-*x*Cu alloys after 3-day co-culture, with blue (nuclei) and red (actin filaments) fluorescence staining. Cells exhibit intact cytoskeletons with polygonal-to-spindle morphologies and clearly visible nuclei, proteins, and pseudopodia, indicating healthy osteoblast growth and high activity. Figure 7B shows live/dead staining of osteoblasts on Ti-*x*Cu alloy surfaces. The viability of osteoblasts exceeded 90% on all Ti-*x*Cu groups (Figure 7C), with no statistically significant differences observed among groups. The CCK-8 assay demonstrated significantly increased osteoblast proliferation in Cu-containing groups after 5 days of co-culture (Figure 7D). At day 7, the Ti-6Cu group exhibited superior proliferation compared to other groups. These results indicate that Ti-*x*Cu alloys exert no significant inhibitory or cytotoxic effects on osteoblasts, with the Ti-6Cu alloy showing optimal cytocompatibility.

### 3.5 Response of osteogenesis to Ti*-x*Cu alloys

**FIGURE 8:**
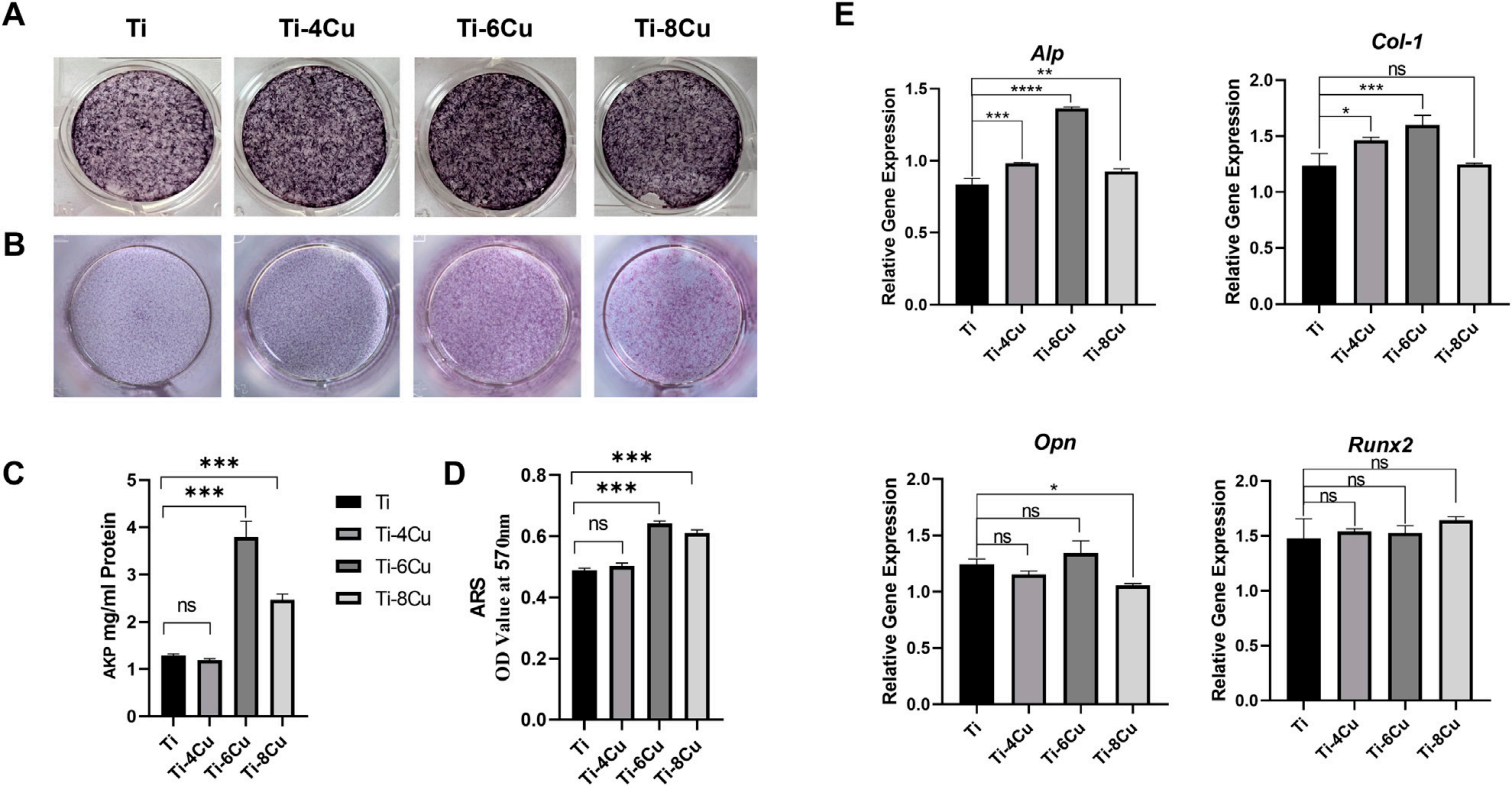
**(A)** The ALP staining of osteoblasts cultured in Ti-*x*Cu alloy extracts for 7 days, and **(C)** the corresponding statistical result; **(B)** the ARS staining of osteoblasts cultured in Ti-*x*Cu alloy extracts for 14 days; **(D)** the corresponding statistical result; **(E)** the expression of osteogenic genes in cells cultured with Ti-*x*Cu alloys.

Figure 8A shows the alkaline phosphatase (ALP) staining results of osteoblasts cultured in Ti-xCu alloy extracts for 7 days. In the 6Cu and 8Cu groups, the samples displayed progressively larger clusters of purple calcium nodules, with the Ti-6Cu group exhibiting the most intense staining (*P < 0.05* vs. other groups, Figure 8C), indicating higher ALP expression levels. Figure 8B presents Alizarin Red S (ARS) staining of osteoblasts cultured in Ti-*x*Cu alloy extracts for 14 days. The Ti-6Cu and Ti-8Cu groups demonstrated significantly higher ARS staining intensity than other groups (*P < 0.05*), suggesting enhanced extracellular matrix mineralization (Figure 8D). To further ascertain the impact of Cu in titanium alloys on osteogenic differentiation, RT-PCR was utilized to evaluate the expression of osteogenic genes in cells cultured with Ti-*x*Cu alloys, the expression of the alkaline phosphatase (*Alp*) gene in Figure 8E indicates that on Ti-4, 6, 8 Cu groups had higher expression, and Ti-6Cu had the highest performance (*P* < 0.05), indicating enhanced early osteogenic activity. Collagen type 1 (*Col-1*) expression was increased in the Ti-4Cu and Ti-6Cu groups, indicating enhanced collagen synthesis, osteoblast differentiation, and bone matrix mineralization. These findings suggest that optimal levels of Cu content in pure Ti, especially in the Ti-6Cu, can effectively promote osteogenic differentiation, making it a potential candidate material for bone tissue engineering.

### 3.6 Response of macrophage to Ti-*x*Cu alloys

**FIGURE 9:**
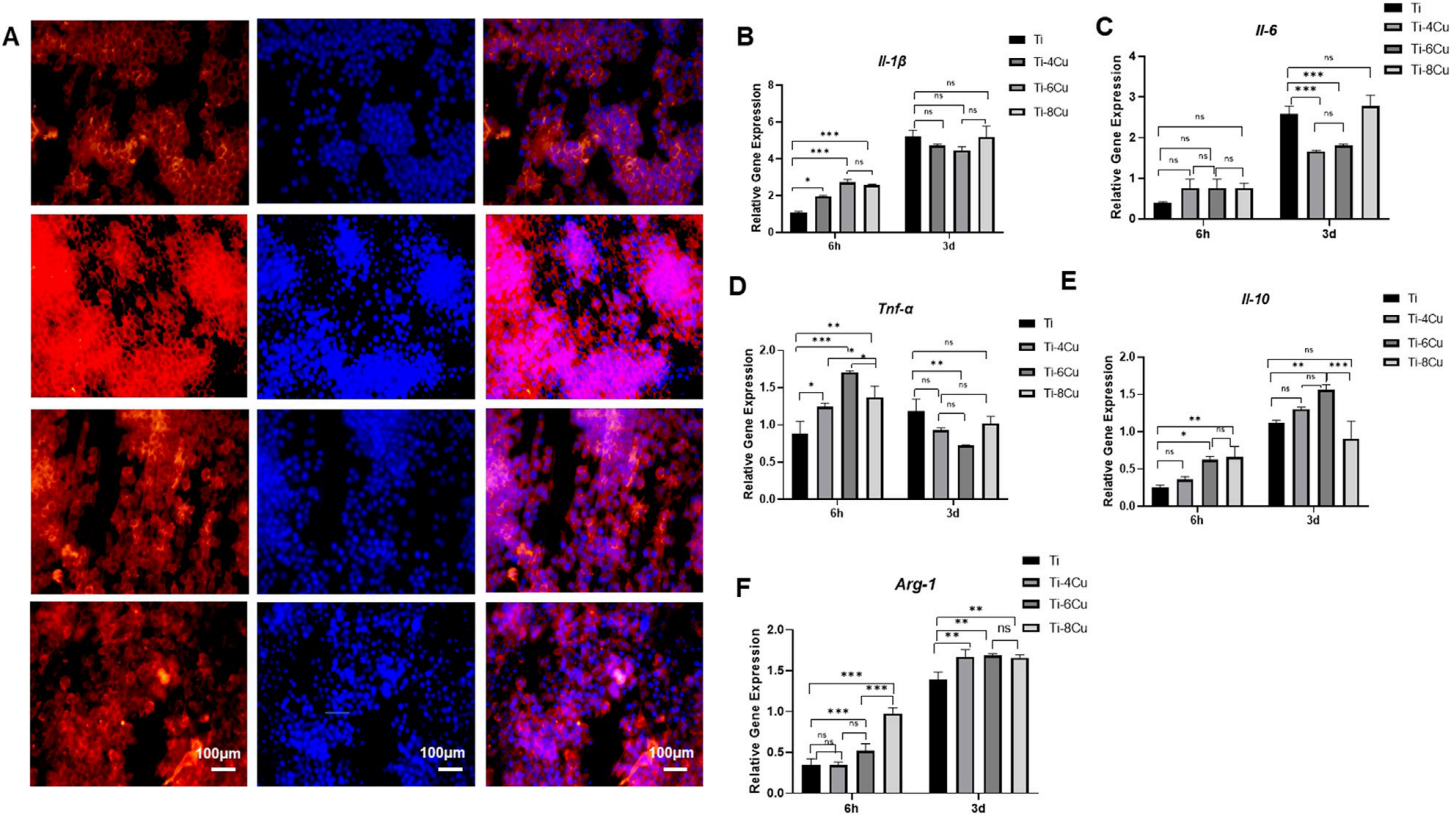
**(A)** Fluorescent images of RAW264.7 cultured on Ti-*x*Cu alloy for 3 days with actin stained with rhodamine phalloidin (red) and nuclei stained with DAPI (blue); **(B**–**F)** The expression of pro-inflammatory and anti-inflammatory cytokines following macrophage culture with Ti-*x*Cu for 6 h and 3 days.

Figure 9A depicts the morphology of RAW264.7 on Ti-*x*Cu alloy after 3 days of co-culturing to assess the production of inflammatory and anti-inflammatory cytokines following macrophage culture with Ti, Ti-4Cu, Ti-6Cu, and Ti-8Cu for 6 h and 3 days. The findings revealed a greater number of star-shaped macrophages on the surfaces of the copper-enriched titanium alloys, Ti-6Cu and Ti-8Cu, as opposed to those on Ti-4Cu. Figures 9B–F depict the levels of inflammatory cytokines (*Il-1β*, *Il-6*, and *Tnf-α*) and anti-inflammatory cytokines (*Il-1 and, Arg-1*) in macrophages cultured on Ti-*x*Cu alloys at 6 h and 3 days. Initially, at the 6-h mark, there is an upregulation of inflammatory cytokines *Il-1β* and *Tnf-α*, particularly in Ti-6Cu, indicating an early inflammatory response. After 3 days of culture, the expression of *Tnf-α* decreases. Regarding the anti-inflammatory cytokines *Il-10* and *Arg-1*, the titanium alloys containing copper generally tend to increase the expression levels of these cytokines at all observed time points. However, it is important to note that there is an exception for Ti-8Cu, compared to Ti-6Cu, where a decrease in *IL-10* expression is observed. This shift suggests that Cu-bearing Ti alloys, especially those with higher Cu content, may initially trigger inflammation but then promote a transition toward an anti-inflammatory and tissue-regenerative state in macrophages. It is known that the elongation of macrophages can boost the M2 subtype’s capacity to produce anti-inflammatory cytokines and shield cells against stimulation by interferon-gamma and lipopolysaccharide. Consequently, the copper-containing titanium alloys, Ti-6Cu and Ti-8Cu, may have the ability to stimulate the polarization of macrophage RAW264.7 cells, offering a potential advantage over the Ti-4Cu alloy.

### 3.7 *In vivo* study

**FIGURE 10:**
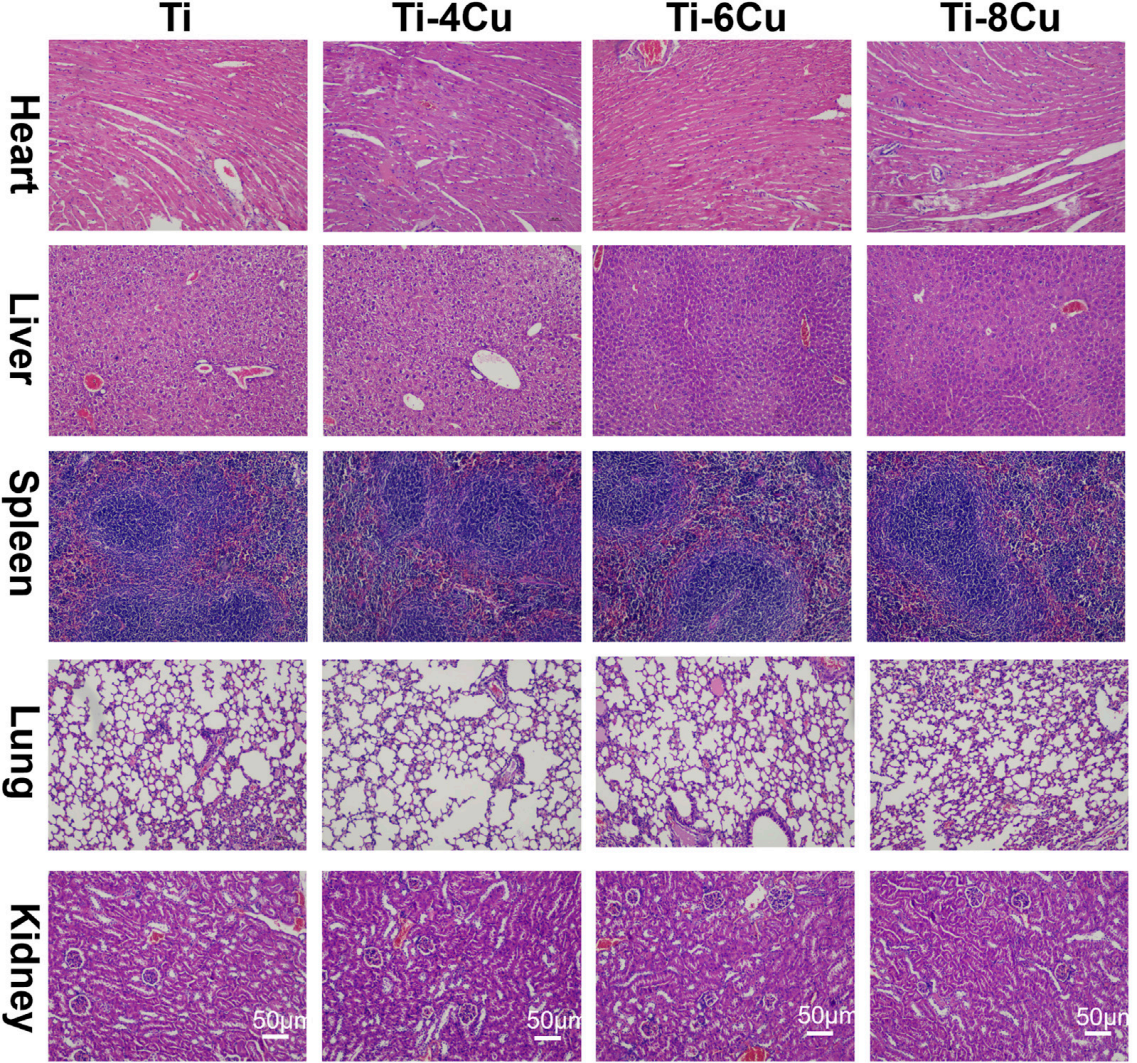
H&E staining of the heart, liver, spleen, lungs, and kidneys after a 7-day implantation in mice.

The *in vivo* biocompatibility of Cu-bearing Ti alloys was tested through animal trials. After a 7-day implantation period in mice, the biosafety of the heart, liver, spleen, lungs, and kidneys was assessed using H&E staining, as shown in Figure 10. Histological analysis revealed preserved cellular architecture across all examined tissues. Cardiomyocytes exhibited regular fiber alignment without structural damage, interstitial congestion, or inflammatory infiltration. Hepatic tissue maintained normal lobular organization with intact sinusoids, devoid of congestion or hemorrhage. Splenic lymphocytes displayed consistent density and clear red–white pulp demarcation. Pulmonary histology showed thin alveolar septa without inflammatory exudates or hemorrhage. Renal tissues demonstrated normal tubular epithelium and glomeruli, absent degenerative changes or interstitial inflammation. The outcomes show no significant histopathological changes in the tissue sections analysis, which suggests that the Ti-4Cu, Ti-6Cu, and Ti-8Cu alloys have excellent *in vivo* compatibility and release ions at safe levels that do not cause organ toxicity.

## 4 Discussion

Titanium meshes are widely utilized as scaffolds in guided bone regeneration; however, their inherent lack of antibacterial properties renders them susceptible to infection, particularly in cases of postoperative wound exposure. To address this limitation, this study introduced copper (4 wt%, 6 wt%, and 8 wt%) into pure titanium to fabricate Ti-Cu alloys using selective laser melting (SLM). While Ti-Cu alloys are promising, systematic investigations into how varying Cu content modulates their microstructure and biological performance remain limited. Therefore, this work aimed to comprehensively evaluate the effects of Cu content on the corrosion resistance, biocompatibility, osteogenic potential, and immunomodulatory properties of these SLM-fabricated alloys. Our findings demonstrate that the Ti-6Cu alloy, in particular, exhibits an optimal balance of properties, including robust antibacterial activity, favorable biosafety, and the ability to suppress inflammatory responses while enhancing osteogenic gene expression.

A critical aspect of these alloys is their mechanism of ion release. Immersion tests revealed that the concentration of released Cu ions increased proportionally with the alloy’s Cu content. This phenomenon is not indicative of deteriorating bulk corrosion resistance but is instead attributed to microgalvanic coupling between the intermetallic Ti_2_Cu phase and the α-Ti matrix. Within these microgalvanic cells, the Ti_2_Cu phase acts as the anode and the subsequent release of Cu ions. This interpretation is supported by electrochemical tests, which confirmed that the overall corrosion resistance was not compromised. This can also be confirmed by the electrochemical tests, which indicate that increasing Cu tends to decrease corrosion current density and R_p_ value.

Notably, a detailed analysis of corrosion behavior revealed nuances related to Cu concentration. The slight increase in corrosion current density (Icorr) for Ti-8Cu versus Ti-6Cu arises from microgalvanic effects between Ti_2_Cu precipitates and the α-Ti matrix. At 8 wt% Cu, the enlarged Ti_2_Cu volume fraction and size (Figure 4D) create more extensive α-Ti/Ti_2_Cu interfaces, forming local galvanic couples where anodic Ti_2_Cu preferentially corrodes. Moreover, larger precipitates disrupt passive film continuity, providing more defect sites for chloride penetration. Although titanium’s inherent passivation maintains acceptable corrosion resistance, the increased microgalvanic activity at higher Cu content elevates Icorr, consistent with reports of optimal corrosion resistance at intermediate Cu concentrations, balancing antibacterial efficacy and passive film stability.

The antibacterial properties of copper-containing alloys are attributed to two primary mechanisms: the release of cytotoxic Cu^2+^ ions and direct contact killing. The ion-release mechanism involves Cu^2+^ penetrating the bacterial cell membrane, where it binds to thiol groups, leading to protein denaturation and the disruption of critical cellular processes such as respiration and substance transport (Mahmoudi et al., 2022). The efficacy of this mechanism is highly dose-dependent. For instance, Liu et al. (2014) reported that Ti-Cu alloys required at least 5 wt% Cu to achieve stable and significant antibacterial effects. However, excessive Cu content can induce cytotoxicity and compromise the material’s biocompatibility (Zhang et al., 2021). Maintaining local Cu^2+^ concentrations within the range of 40–220 μmol/L is optimal for providing antibacterial benefits and promoting cell proliferation in wound healing (Zhang et al., 2024b). In the present study, the Cu ion concentrations released from our alloys (0.59–0.94 mg/L ≈ 9.3–14.8 μmol/L) fell below this optimal range, suggesting that ion release alone is insufficient to account for the potent bactericidal activity observed.

Currently, a contact killing mechanism is considered the main significant antibacterial pathway for Ti-Cu alloys. That is because Cu in its solid solution form does not show improved antibacterial performance (Mahmoudi et al., 2022). Guo et al. (2017) demonstrated that SLM-fabricated Ti6Al4V-Cu alloys exhibit Cu-content-dependent antibacterial efficacy, achieving about 99% microbial reduction at >4 wt% Cu. In this study, when the Cu content was higher than 4 wt%, Ti-4Cu, Ti-6Cu, and Ti-8Cu exhibited increased antibacterial capabilities, consistent with Guo et al.'s finding. This indicates that the presence of Cu ions in solution is not the primary factor in killing bacteria. Substantial evidence indicates that the effectiveness of contact killing is directly proportional to the surface area of the Ti_2_Cu phase available for bacterial interaction (Ji et al., 2021; Xie et al., 2022). Zhang et al. (2016) have confirmed that direct physical contact with the Ti_2_Cu phase on the alloy surface is required to effectively eradicate bacteria. Therefore, the dense, lamellar Ti_2_Cu phase observed in our Ti-Cu alloys with ≥4 wt% Cu (Figure 4) is identified as the primary structural feature responsible for their excellent antibacterial properties. Even when the Cu content in Ti is increased to 8 wt%, the concentration of released Cu ions remains below the effective antibacterial concentration range of localized Cu^2+^, yet the material still shows excellent antibacterial activity. We therefore infer that both ionic and contact-mediated antibacterial mechanisms act synergistically to produce this performance.

The biocompatibility of implant materials must be rigorously assessed prior to *in vitro*/*vivo* studies. In this study, Ti ions released from Ti-*x*Cu in a 0.9 wt% NaCl solution increase with an increased Cu content. In parallel, the Cu ion concentration also shows a steady increase. However, the Cu ion release from the Ti-*x*Cu alloy was below 0.94 mg/L/7d, which is lower than the World Health Organization’s recommended minimum daily copper intake for adults (approximately 1.3 mg/d) (Turnlund, 1988). Cellular adhesion analysis revealed well-defined cytoplasmic extensions and nuclear morphology with extensive spreading across the Ti-4Cu, Ti-6Cu, and Ti-8Cu alloys, suggesting enhanced cellular viability. In the *in vivo* test, Cu-bearing titanium was implanted subcutaneously in the backs of mice for 7 days. Histological examination of HE-stained sections of internal organs showed no abnormalities. These findings suggest that Ti-4Cu, Ti-6Cu, and Ti-8Cu demonstrate an acceptable level of biocompatibility.

Copper-containing metallic materials exhibit excellent performance in promoting osteogenesis and cartilage regeneration (Dang et al., 2018; Wang et al., 2021b). Research has shown that Cu ions enhance the mineralization in osteoblasts and stimulate the expression of vital genes associated with bone formation, such as *Alp*, *Opn*, and *Ocn* (Li et al., 2025; Wu et al., 2023). Li et al. also reported that Ti6Al4V-4.5Cu supports the coupling of angiogenesis and osteogenic (Li et al., 2023). Our previous studies indicate that Cu-enriched titanium alloys exhibit good biocompatibility with gingival fibroblasts and osteoblasts, while demonstrating anti-inflammatory effects on macrophages and pro-angiogenic potential. These properties support their application in titanium alloy meshes for alveolar bone regeneration (Xu et al., 2018). Therefore, the osteogenic impact of copper ions might be contingent upon the duration of observation, varying mass fractions of copper, and distinct preparation methods. In this study, following a 7-day co-culture of Ti-*x*Cu with osteoblasts, the Ti-4Cu and Ti-6Cu samples exhibit increased *Col-1* gene expression levels that rose with increasing Cu concentrations. Additionally, the Ti-6Cu group increases the expression of *Alp*, a gene integral to bone formation. *Alp* and *Col-1* serve as markers for the early stages of osteoblast differentiation. The findings suggest that Ti-Cu alloys containing less than 8 wt% Cu can stimulate early cell adhesion, *Alp* activity, and the expression of genes associated with osteogenic differentiation, thereby facilitating osteogenic differentiation, with the Ti-6Cu variant demonstrating superior osteogenic properties.

The host immune response, particularly the activity of macrophages, is a critical determinant of bone regeneration and implant integration (Luo et al., 2019). The modulation of inflammation can either facilitate or hinder wound healing and bone tissue regeneration (Molitoris et al., 2024; Molitoris et al., 2024). Implanting biomaterials that provoke a severe inflammatory response may lead to early resorption, material rejection, and fibrosis around the implant site (Vishwakarma et al., 2016). The incorporation of Cu into the material surface enhances macrophage-mediated osteogenesis and antibacterial efficacy (Huang et al., 2018). Consequently, the modulation of Cu ions’ impact on macrophages has attracted significant interest, given the potential of Cu ions to modulate macrophage responses (Wang et al., 2021a). Activated M1 macrophages demonstrate stimulatory effects on osteoblast maturation and increase bactericidal activity against *S. aureus* (Huang et al., 2019). Although these findings suggest the pro-inflammatory potential of Cu, recent studies have revealed that copper ions exert concentration-dependent effects on macrophage polarization (M1/M2) and activation responses (Díez-Tercero et al., 2021). For example, Xu et al. (2021) discovered that Ti6Al4V-6Cu notably impedes the proliferation of macrophages, diminishes the release of pro-inflammatory genes, and encourages a transition toward the M2 phenotype. Moreover, Lu et al. (2023) suggested that copper-enriched CoCr alloys can mitigate macrophage-driven inflammation and osteoclast differentiation without hindering the osteoblast proliferation. The 3D-printed Ti-5Cu alloy accelerates osteogenic differentiation of MC3T3-E1 cells by stimulating macrophage polarization toward the M2 phenotype (Zhao et al., 2022). In alignment with these findings, our results demonstrate that the Ti-6Cu alloy favorably modulates the macrophage response. As shown in Figure 7, after a 3-day culture, the Ti-6Cu group exhibited significantly reduced expression of pro-inflammatory factors alongside increased secretion of the anti-inflammatory cytokine *Il-10* and upregulation of *Arg-1* expression compared to the pure Ti control. This immunological profile is indicative of a pronounced shift toward M2 macrophage polarization. By fostering an anti-inflammatory and pro-regenerative microenvironment, the SLM-fabricated Ti-6Cu alloy combines excellent antibacterial efficacy with favorable immunomodulatory activity, highlighting its significant potential for clinical applications such as titanium mesh for bone reconstruction.

In summary, SLM-fabricated Ti-6Cu alloys show strong clinical potential for guided bone regeneration (GBR), offering precision and cost efficiency. The SLM technology enables patient-specific titanium meshes with high anatomical accuracy, reducing surgical complications (e.g., mesh exposure) and improving outcomes. While initial SLM costs (equipment/Ti-Cu powders) exceed conventional methods, savings from fewer revisions, infections, and hospitalizations offset the additional cost. The alloy’s intrinsic antibacterial and osteogenic properties further minimize adjunct therapies. Future studies should systematically evaluate the long-term *in vivo* and *in vitro* toxicity of Ti-6Cu alloy, along with its associated biological mechanisms. Future scalability of SLM production and optimization of Cu-containing powder synthesis could further improve cost efficiency, making Ti-6Cu alloys a viable and transformative option for personalized GBR applications.

## 5 Conclusion

In this study, Ti-*x*Cu alloys with Cu contents of 4 wt%, 6 wt%, and 8 wt% were successfully fabricated utilizing the SLM technique. Incorporating Cu into pure titanium resulted in an increased presence of Ti_2_Cu within the matrix, yet this had a negligible effect on the resistance to electrochemical corrosion. The release of Cu and Ti ions was found to correlate positively with the Cu mass within the matrix. All Ti-*x*Cu alloys demonstrated an antibacterial efficacy exceeding 99% against both *E. coli* and *S. aureus*; the lamellar Ti_2_Cu phase is postulated to be the predominant antibacterial component. Notably, Ti alloys containing 4 wt% and 6 wt% Cu demonstrated increased expression of osteogenesis-related genes in MC3T3-E1 cells. The Ti-6Cu alloy could mitigate local inflammation by suppressing macrophage activity, as indicated *in vitro* tests.

Consequently, it is expected that the SLM-manufactured Ti-6Cu alloy is a promising application for titanium mesh, with the capacity to construct alveolar bone. This study provides a foundation for further research into the mechanisms of copper-induced inflammatory responses and osteogenic effects, while offering insights into the development of copper-containing titanium scaffold materials.

## Data Availability

The raw data supporting the conclusions of this article will be made available by the authors, without undue reservation.
